# Non-Operative Treatment of Strabismus

**Published:** 1903-05

**Authors:** 

**Affiliations:** Brownwood, Texas


### Eye, Ear, Nose and Throat.

EDITED BY DR. P. M. PAYNE, BROWNWOOD, TEXAS.

### Non-Operative Treatment of Strabismus.

The *Annals of Ophthalmology* contains the following taken from *Journal of American Medical Association*, November 1, 1902. Dr. Geo. M. Gould, of Philadelphia, says: "In my private practice I have in six years seen no case that I believe needed operation, and I have seen none benefited thereby, at least none that could not have been benefited by other methods of treatment."

"All strabismus is preceded by heterophoria and chronic or permanent strabismus is preceded by a stage of acute, functional or incomplete strabismus." Gould lays down as exceptions, traumatic, paralytic, most cases of alternating and some anomalous cases. "The non-operative treatment of strabismus naturally divides itself into (1) prophalaxis; (2) the treatment of ametropia; (3) the treatment of heterophoria; (4) the treatment of amblyopia; (5) the treatment of physiologically curable strabismus; (6) the treatment of alternating strabismus of anomalous cases." I think every occulist should give the deepest study to this subject, for among the incurable cases he includes those that have been "unsuccessfully operated on" for insufficiency or actual strabismus.

If we would teach our patients the proper use of exercise prisms, for daily practice, correcting the ametropia with proper lenses for constant use instead of "snipping" the strong muscle or advancing the weak one; if we would teach them to cover up the fixing eye and force the squinting eye to perform its function for at least a few moments daily we would achieve more than by an operation which only too often fails of its purpose—that of improving the vision and the patient's personal appearance.

---

"Trachoma Amenable to the X-Ray," is the title of an article in *The Journal of Eye, Ear, Nose and Throat Diseases* of Baltimore, Md., by Drs. Cassiday and Bayne, radiographers to the Maryland General Hospital.

They describe the process minutely, then ask the question: "How does the X-Ray produce such results?" "Probably it is a neuropathic effect, in which normal tissues are stimulated to new vigor, and pathological tissue, always being of less resistance, assumes a retrograde metamorphosis." If this proves a permanent success, it will be a blessing to eye clinics for there trachoma is the bete noir.

---

THE "ACOUSTICON"—AN INSTRUMENT WITH WHICH THE DEAF HEAR PERFECTLY.—The lay press has been having much to say of the "Acousticon," an instrument invented by a Mr. Hutchinson, of Alabama. He is said to have demonstrated the value of his invention in Buckingham Palace and to have received a gold medal from Queen Alexander in recognizition of his services to science.

